# Muscle oxygen saturation plateau: Definition and verification in different oxygen availability conditions in locomotor and non‐locomotor muscles

**DOI:** 10.1113/EP092862

**Published:** 2025-07-29

**Authors:** Tomasz Kowalski, Kinga Rębiś, Adrian Wilk, Dominika Granda, Andrzej Białecki, Tadej Debevec, Raphael Faiss

**Affiliations:** ^1^ Department of Physiology Institute of Sport—National Research Institute Warsaw Poland; ^2^ Department of Nutrition Physiology Institute of Sport—National Research Institute Warsaw Poland; ^3^ Faculty of Electronics and Information Technology Warsaw University of Technology Warsaw Poland; ^4^ Faculty of Sport University of Ljubljana Ljubljana Slovenia; ^5^ Department of Automatics, Biocybernetics and Robotics ‘Jozef Stefan’ Institute Ljubljana Slovenia; ^6^ Institute of Sports Sciences, Faculty of Social and Political Sciences University of Lausanne Lausanne Switzerland

**Keywords:** critical oxygenation, critical power, elite athlete, exercise, hypoxia, near‐infrared spectroscopy

## Abstract

Novel wearable near‐infrared spectroscopy devices allow for a better understanding of muscle oxygenation kinetics during exercise. A muscle oxygen saturation (SmO_2_) plateau is often applied in the scientific literature, but clear criteria for its definition remain unestablished to date. The aim of this study was to develop criteria allowing for definition of SmO_2_ plateaus. Multiple variables associated with physiological plateaus during exercise were assessed to develop a framework for an SmO_2_ plateau. Subsequently, the existence of an SmO_2_ plateau during 3 min all‐out cycling trials (critical oxygenation plateau) was tested in different conditions of oxygen availability (i.e., normoxia and hypoxia) in vastus lateralis and triceps brachii among 30 endurance athletes. Plateau determination methods based on a threshold of change of ±5 arbitrary units (a.u.) of SmO_2_ and expert visual assessment showed almost perfect agreement. However, a threshold of 10 a.u. yielded high SmO_2_ variability associated with a large number of possibly false‐positive detections. Conversely, relative changes (thresholds of 5% and 10%) did not align with other methods, corresponding to low absolute changes, limiting their applicability. The inter‐rater agreement between individual visual assessments exhibited a higher reliability among expert versus non‐expert raters. The determination of an SmO_2_ plateau depends on the applied methodology. Overall, a critical oxygenation plateau was observed in the vastus lateralis in both normoxia and hypoxia in >90% of cases. The results of this study allow recommendation either for the use of a threshold of change corresponding to 5 a.u. of SmO_2_ or expert visual assessment, using 30 s segments.

## Abstract (highlights)

- **What is the central question of this study?**
  What is the muscle oxygen saturation plateau, and how should it be defined?
- **What is the main finding and its importance?**
  The determination of the muscle oxygen saturation plateau depends on the methodological approach applied. This plateau can be determined effectively using a threshold of change corresponding to 5 arbitrary units of percentage muscle oxygen saturation or expert visual assessment. Application of the recommended criteria, along with transparent reporting, might enhance the rigour of studies examining muscle oxygenation kinetics.

## 1 INTRODUCTION

Critical power (CP) represents the highest sustainable power output that an individual can maintain over a given period without fatigue‐induced failure. It represents the boundary between heavy and severe exercise domains, and between steady‐state and non‐steady‐state exercise intensity (Jones et al., 2008; Poole, 2009). The CP, with its sport‐specific variants (e.g., critical speed for swimming or running), has exhibited noteworthy scientific and practical utility and remained the object of thorough research for decades (Hill, 1925; Monod & Scherrer, 1965; Vanhatalo et al., 2011). Classically, multiple efforts were required to establish CP, typically involving several exhaustive trials at different intensities. More recently, the single‐effort 3 min all‐out test has become very popular owing to its feasibility, simplicity and reliability. In this test, participants perform an all‐out effort for 3 min without deliberate pacing (i.e., with a maximal effort from the start). In such settings, power output reaches a plateau in the final phase after ∼125 s of effort (Vanhatalo et al., 2007). This stabilized power represents CP and is associated with a maximal aerobic steady state (Chorley & Lamb, 2020).

Although CP is strongly correlated with multiple physiological variables, it is based on performance and externally measured mechanical work (Jones et al., 2010). Consequently, many authors attempted to fit CP into the exercise physiology framework (Broxterman et al., 2015; Jones et al., 2019). A detailed explanation of the physiological and mechanistic background of CP is beyond the scope of this paper, but it is widely associated with the highest steady‐state oxidative metabolism rate during exercise (Jones et al., 2010). Therefore, the hyperbolic power–duration curve defines the limit of exercise at specific durations and plateaus at CP (Poole et al., 2016). Such a plateau was also observed after 125 s of a 3 min all‐out effort (Vanhatalo et al., 2007). It was suggested that a corresponding plateau, associated with muscle oxygen availability, exists and represents a steady state in local oxygen supply and demand. Given that local muscle oxygen saturation (SmO_2_) mirrors systemic CP, a ‘critical oxygenation’ (COx) variable was proposed (Feldmann & Erlacher, 2021). With the development of wearable near‐infrared spectroscopy (NIRS) devices, SmO_2_ can be measured in a non‐invasive way, allowing for effective and convenient identification of the steady state in local oxygenation (Matthews et al., 2023).

Although the concept of an SmO_2_ plateau is applied in the scientific literature (Barstow, 2019; Feldmann & Erlacher, 2021), it has never been precisely defined or properly validated. Contrary to multiple physiological variables, such as maximum oxygen uptake ($\dot{V}O_{2}\max$) or maximum lactate steady state (MLSS) (Bassett & Howley, 1997; Billat et al., 2003), no criteria for SmO_2_ plateau are currently established. Multiple studies have indicated that muscle oxygenation exhibits a sigmoidal pattern, with the SmO_2_ asymptote or plateau occurring near the end of the incremental step and ramp tests to exhaustion (Boone et al., 2010; Racinais et al., 2014). It was reported that an oxygenation plateau was observed in the vastus lateralis in 12 of 14 subjects during incremental cycling in normoxia (Ferreira et al., 2007). Likewise, SmO_2_ profiles at both vastus lateralis and lateral deltoid muscles displayed a near‐plateau or breakpoint response near the respiratory compensation point during the incremental ramp cycling test in normoxia (Yogev et al., 2022). However, deoxygenation patterns at intensities exceeding the threshold of 75% of peak power from a ramp test to exhaustion were observed to vary significantly in different oxygen availability conditions. In the study from Subudhi et al. (2007) addressing vastus lateralis SmO_2_ during a cycling ramp test, all individuals reached an SmO_2_ plateau at >90% of the peak power in normoxia, but fewer than half demonstrated such a plateau in acute hypoxia of ∼4300 m a.s.l. (Subudhi et al., 2007).

Constant‐load tests at submaximal intensity typically exhibited a SmO_2_ plateau. Moreover, SmO_2_ was relatively constant for both steady‐state intensity and the workload slightly above this intensity during MLSS running assessment in trained endurance athletes (Snyder & Parmenter, 2009). Stable SmO_2_ in vastus lateralis during constant‐load cycling in normoxia performed by trained cyclists at both 75% and 85% of peak power from an incremental test to exhaustion was also observed (Oueslati, 2018). Hopker et al. (2017) applied a 2 h cycling constant‐load test in a moderate intensity domain in normoxia. They observed a trend of vastus lateralis SmO_2_ reduction across the effort; however, the differences were not statistically significant (approximately 2–3 a.u. of %SmO_2_; Hopker et al., 2017). Similar SmO_2_ values measured on quadriceps across 30 min of aerobic cycling effort were reported for both normoxia and hypoxia (i.e.,, targeted arterial oxygen saturation of 75%; Chacaroun et al., 2019). Likewise, in our recent study addressing MLSS running efforts in trained ski‐mountaineers, SmO_2_ plateaus were observed at sea level and the simulated altitude of 3000 m a.s.l. (Rębiś et al., 2024). Overall, the criteria for SmO_2_ plateau were rarely presented in the discussed studies, and the observations were focused predominantly on large locomotor muscle groups and exercise in normoxia.

The aim of this study was therefore to clarify the concept of an SmO_2_ plateau. Specifically, we aimed: (1) to analyse other well‐established variables that are associated with some kind of physiological plateaus to develop a framework allowing for defining and determining an SmO_2_ plateau; and (2) to verify the existence of COx plateaus in different conditions of oxygen availability in vastus lateralis and triceps brachii among highly trained endurance athletes.

## 2 MATERIALS AND METHODS

The study protocol received ethical approval from the Institute of Sport—National Research Institute Ethics Committee, Warsaw, Poland (approval no. KEBN‐24‐97‐KR). All procedures adhered to the principles outlined in the *Declaration of Helsinki*, and informed written consent was obtained from all participants prior to their involvement in the study.

### 2.1 Rationale for framework determination

Apart from CP, $\dot{V}O_{2}\max$ and MLSS play important roles in exercise physiology and sports science, because they integrate and reflect the function of various physiological systems (Beneke, 2003; Montero et al., 2015). They are also traditionally associated with plateaus of physiological variables and are often considered (not without controversy) to demarcate exercise intensity domains (Jamnick et al., 2020).

Mathematically, a plateau is defined as a segment of a function where the slope is zero. However, oxygen‐uptake values from successive sampling intervals never match exactly, even in steady‐state conditions (Myers et al., 1990). When the breath‐by‐breath sampling method is used, the irregularities in oxygen uptake come from variability in breathing frequency and depth. Likewise, certain fluctuations are observed across lactate measurements and in cycling power analyses during exercise (Pfitzinger & Freedson, 1998; Jeffries et al., 2019). In a seminal work from Vanhatalo et al. (2007), repeated‐measures ANOVA was used to compare power output across 15 s averages during a 3 min all‐out effort, and the plateau was associated with a lack of significant changes between the segments (Vanhatalo et al., 2007). The $\dot{V}O_{2}\max$ is conventionally defined by the presence of a plateau in oxygen consumption despite increasing exercise intensity (Bassett & Howley, 1997). Although there is significant variability in how studies define the plateau, popular values are ≤150 mL/min or <2% increase in absolute oxygen uptake despite increasing workload [Liguori & American College of Sports Medicine (ACSM), 2021]. Interestingly, the guidelines rarely specify an exact duration for the plateau in oxygen uptake. Typically, samples from 15–30 s are applied (Astorino, 2009). The MLSS plateau indicates that lactate production and clearance are balanced, typically observed when blood lactate levels fluctuate by no more than 1.0 mmol/L between the 10th and 30th minute of exercise (Heck & Wackerhage, 2024). Some studies use 0.5 mmol/L as a more conservative criterion, whereas others explore variations ≤1.5 mmol/L, depending on the protocol and investigated population (Llodio et al., 2024; Urhausen et al., 1993). In summary, different approaches to defining and establishing physiological plateaus are applied in exercise physiology.

The NIRS signal from biosensors is also characterized by significant variability owing to physiological and technical factors (Barstow, 2019). For example, the default setting of Moxy firmware, which is a NIRS device used in the present study, smoothes the signal with a 5 s moving average (Moxy Monitors, Hutchinson, MN, USA). In addition to biological heterogeneity, movement artefacts, haemodynamic shifts and changes in skin perfusion during intense exercise pose challenges to signal reliability. Therefore, when we define an SmO_2_ plateau, we are really defining a narrow range of acceptable measurement variability that is not associated with significant changes. Based on discussions within the research team, in frameworks already used for CP, $\dot{V}O_{2}\max$ and MLSS, multiple absolute and relative variability thresholds were suggested and applied in this study. Additionally, the visual method was proposed. Like the accepted gold‐standard methodology of ventilatory threshold evaluation during cardiopulmonary exercise testing, it should be conducted by multiple skilled reviewers (Zignoli et al., 2022). Such an approach was already exhibited to confirm the inflection point in the NIRS‐derived SmO_2_ profile plotted against time during incremental ramp testing (Yogev et al., 2022).

### 2.2 Participants

Data from 120 samples from 30 highly trained endurance athletes (10 females) were included in the analyses. Based on the Participant Classification Framework (McKay et al., 2022), the participants were categorized as Tier 5 or Tier 4 athletes, representing world‐class or international‐level competitors (McKay et al., 2022). Inclusion criteria comprised excellent health, world‐class or international‐level performance status in endurance sports, and prior experience with CP testing. Exclusion criteria included any chronic medical conditions, acute illnesses within the past month, ongoing medication use, pregnancy, or exposure to hypoxia in the last 6 months. The participants were recruited through convenience sampling in collaboration with national sports federations and independent elite coaches. All the participants were Caucasian with light or medium skin tones, according to the visual assessment with the Monk Skin Tone Scale (Monk, 2023). Initially, 33 athletes completed all the necessary assessments. However, owing to data‐management issues, samples from three athletes were not taken into account. The basic characteristics of the included participants are presented in Table 1. The seca 274 (seca GmbH & Co. KG, Hamburg, Germany) free‐standing digital stadiometer was applied to measure height. The InBody 770 (InBodyUSA, Cerritos, CA, USA) body composition analyser was used to measure body mass. The adipose tissue thickness at the sensor sites was measured by skilled and experienced staff members using Harpenden Skinfold Calipers (Baty International, Burgess Hill, UK). The $\dot{V}O_{2}\max$ was established during breath‐by‐breath cardiopulmonary exercise testing performed with Cortex Metamax B3 (Cortex Biophysik GmbH, Leipzig, Germany) and Cyclus II Ergometer (RBM, Leipzig, Germany) according to the American College of Sports Medicine guidelines [Liguori & American College of Sports Medicine (ACSM), 2021].

**TABLE 1:** Basic characteristics of participants.

Variable/group	Females (*n* = 10)	Males (*n* = 20)
Age, years	19.9 ± 3.0	22.1 ± 5.9
Body height, cm	166.4 ± 7.5	180.6 ± 5.8
Body mass, kg	57.9 ± 8.3	74.1 ± 9.4
ATT at vastus lateralis, mm	12.2 ± 2.7	5.6 ± 1.6
ATT at triceps brachii, mm	11.2 ± 3.2	5.6 ± 1.7
$\dot{V}O_{2}\max$, mL/kg/min	56.4 ± 4.0	63.0 ± 4.1

*Note*: Values are the mean ± SD.

Abbreviations: ATT, adipose tissue thickness; $\dot{V}O_{2}\max$, maximum oxygen uptake.

### 2.3 Study design

Over the course of 3 days, participants completed two 3 min CP cycling tests in a randomized order: one in normoxia (87 m a.s.l., fraction of inspired O_2_ = 20.8%) and one in normobaric hypoxia (3200 m a.s.l., fraction of inspired O_2_ = 14.2%). To ensure blinding, both tests took place inside a hypoxic chamber (Air Sport, Międzyzdroje, Poland) with placebo mode available. Thus, the participants were not informed whether normoxic or hypoxic conditions were applied. Temperature (∼19°C), humidity (∼50%) and concentrations of oxygen and carbon dioxide were regulated centrally and maintained at constant levels. All the measurements were held at the Institute of Sport—National Research Institute in Warsaw, Poland. All participants were already familiar with CP testing and were instructed to avoid intense training or long‐distance travel for 48 h before assessment. Prior to testing, they underwent a comprehensive medical evaluation, including a resting ECG and blood‐based haematological screening. Upon arrival at the laboratory, all the measurement devices were mounted. Next, the participants remained seated for 30 min while the laboratory technician prepared their bikes on a trainer; each athlete used their personal training or racing bike during the assessment. The warm‐up consisted of 15 min of light‐to‐moderate pedalling (rating of perceived exertion 2–4 on the Borg 1–10 scale), incorporating two 6 s sprints at self‐selected intensity. Immediately before testing, participants were instructed to give a maximal, all‐out effort for the full 3 min duration without pacing. Cadence and body position (seating vs. standing on the pedals) were self‐selected by the participants. Strong verbal encouragement was provided throughout the effort to maximize the performance.

The SmO_2_ was assessed using two Moxy monitors (Moxy Monitors, Hutchinson, MN, USA). These compact, cost‐effective and wearable continuous‐wave NIRS sensors provide an arbitrarily scaled measure of total haem volume [in arbitrary units (a.u.)], representing both haemoglobin and myoglobin concentrations in the illuminated tissue, along with SmO_2_ levels ranging from 0% to 100% (Feldmann et al., 2019; Yogev et al., 2023). The device uses four near‐infrared light wavelengths (680, 720, 760 and 800 nm) to measure tissue oxygenation. The sensor is equipped with a single LED emitter and two photodetectors positioned at distances of 12.5 and 25.0 mm from the light source. The Moxy monitor provides real‐time data on fluctuations in total haem (+myo)globin concentration and the tissue saturation index, reflecting local oxygenation dynamics (McManus et al., 2018). One sensor was positioned on the right vastus lateralis, ∼13 ± 2 cm above the proximal edge of the patella and 4 ± 2 cm lateral to the midline of the thigh. The second sensor was placed on the lateral head of the right triceps brachii, about halfway between the acromion and olecranon. To minimize signal artefacts from movement or ambient light, both sensors were secured using the manufacturer‐supplied light shield and custom‐made adhesive tapes. For all the participants, the tissue thickness remained <15 mm, a threshold necessary for reliable NIRS measurements (Feldmann et al., 2019).

### 2.4 Plateau criteria and statistical analyses

For the purpose of further analysis, the COx plateau was defined as the stable SmO_2_ for 30 s during the last 45 s of the effort within 3 min CP tests.

Based on the aforementioned framework, three different plateau determination approaches were examined on the dataset consisting of 120 files with NIRS signal from 30 athletes. Given that exploratory analysis did not exhibit distinct oxygenation patterns in males and females, further analysis is presented for both sexes combined. The results are provided for locomotor (vastus lateralis) and non‐locomotor (triceps brachii) muscles and for normoxia and hypoxia. Numbers and percentages for COx plateaus observed in different settings with different methods were reported. The following approaches were applied:

Absolute values, i.e., thresholds of change corresponding to 5 (values: −5 to +5) and 10 (values: −10 to +10) SmO_2_ a.u. during 30 s within the last 45 s of the effort;

Relative values, i.e., threshold of change corresponding to 5 (values: −5% to +5%) and 10% (values: −10% to +10%) of SmO_2_ during 30 s within the last 45 s of the effort; and

Visual assessment, i.e., binary evaluation regarding the existence of the SmO_2_ plateau with a visual inspection of the SmO_2_ curve by: (a) four independent exercise physiologists familiar with NIRS analysis (expert group); and (b) four independent, highly trained athletes not familiar with NIRS analysis (non‐expert group). Samples of 4 min (30 s before the test, 3 min test, 30 s after the test) were used to prepare graphs subsequently presented to the raters for evaluation in randomized order. The raters received basic instructions and were blinded to muscle group, sex and oxygen availability conditions. Fleiss’ kappa (κ) rater agreement analysis was performed to examine the reliability of the approach. During the comparative analysis, the positive agreement of at least three of four raters regarding plateau existence was included as confirmation of the plateau for the visual assessment.

**FIGURE 1:**
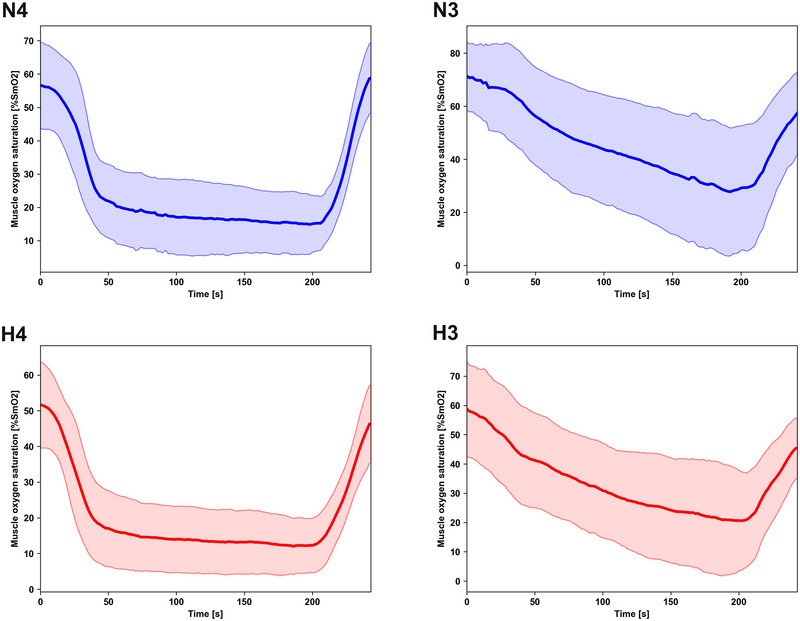
Average SmO_2_ kinetics 30 s before, during, and 30 s after critical power tests for vastus lateralis and triceps brachii in normoxia and hypoxia (*n* = 120 total, *n* = 30 for each category). Values are the mean (lines) and SD (clouds). Panel N4 presents results for vastus lateralis in normoxia. Panel N3 presents results for triceps brachii in normoxia. Panel H4 presents results for vastus lateralis in hypoxia. Panel H3 presents results for triceps brachii in hypoxia. Abbreviations: SmO_2_, muscle oxygen saturation; %SmO_2_, arbitrary unit of muscle oxygen saturation.

**FIGURE 2:**
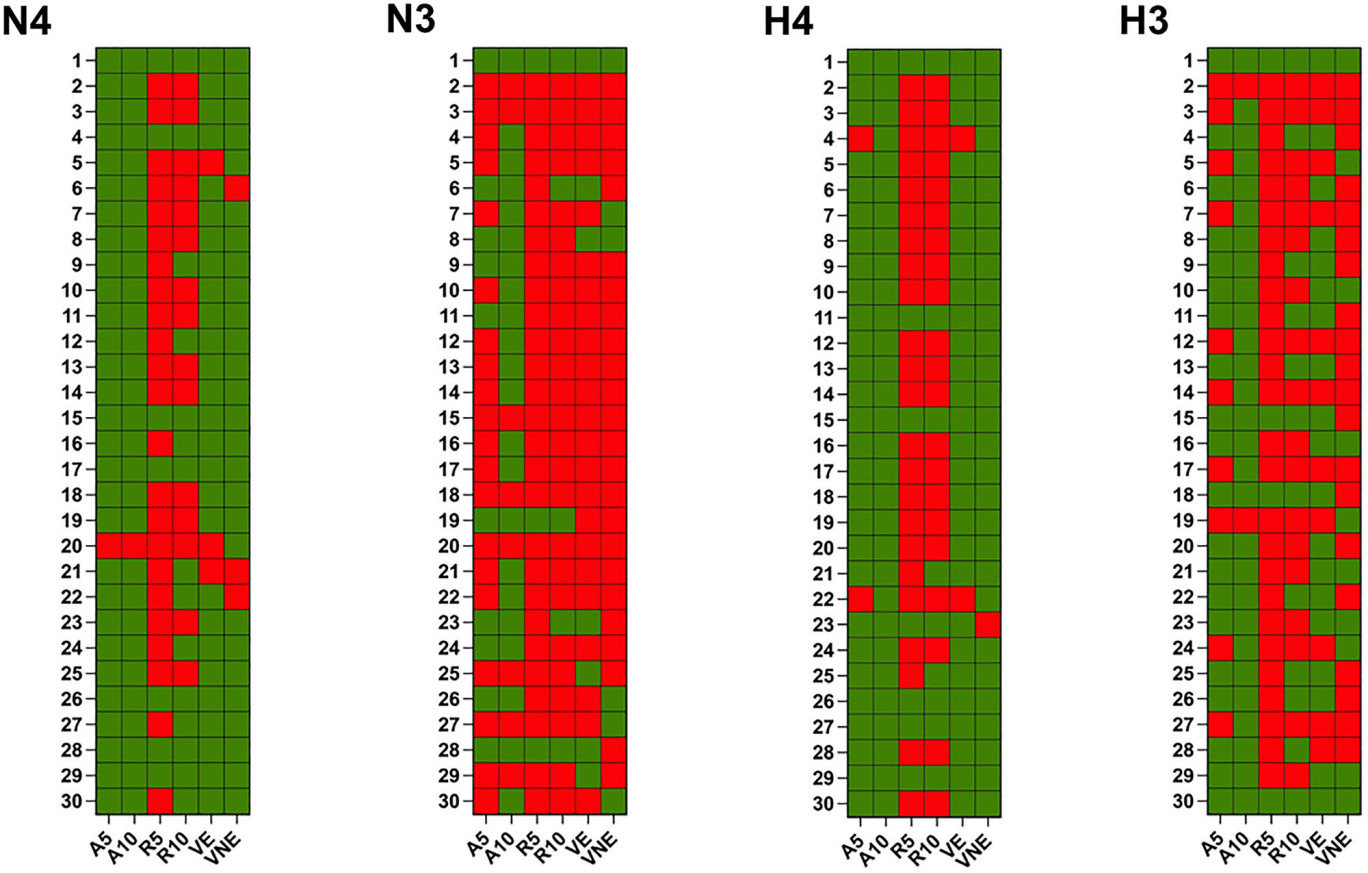
Summary of results for binary COx plateau determination within different methods. Green denotes positive determination of the COx plateau, and red denotes a lack of positive determination of the COx plateau. Panel N4 presents results for vastus lateralis in normoxia. Panel N3 presents results for triceps brachii in normoxia. Panel H4 presents results for vastus lateralis in hypoxia. Panel H3 presents results for triceps brachii in hypoxia. Abbreviations: A5, threshold of change corresponding to 5 a.u. of %SmO_2_ (absolute change); A10, threshold of change corresponding to 10 a.u. of %SmO_2_ (absolute change); a.u., arbitrary units; COx, critical oxygenation; R5, threshold of change corresponding to 5% of %SmO_2_ (relative change); R10, threshold of change corresponding to 10% of %SmO_2_ (relative change); VE, visual assessment of the expert group; VNE, visual assessment of the non‐expert group.

The NIRS data were sampled in 2 s intervals and smoothed with a 5 s moving average, because this is the default setting in the dedicated Moxy firmware (v.1.05.05, Moxy Monitors, Hutchinson, MN, USA). Then, the exported .csv files were adjusted for 4 min samples in Microsoft Excel (v.16.0.5491.1000, Microsoft Corporation, Redmond, WA, USA). Further analyses were performed in Python (v.3.13), including extrapolation of the data to obtain data points from every second and use of the criteria for COx plateau determination presented above (absolute and relative thresholds of change). Detailed descriptions of the applied protocols and Python scripts used in this study are provided in the GitHub repository: (https://github.com/Kaszanas/SmO2_plateau_detection). The agreement between different methods is presented as a percentage. The spm1d Python library was used to prepare Figure 1, and GraphPad Prism (v.10.4.1, GraphPad Software, Boston, MA, USA) to prepare Figure 2.

## 3 RESULTS

Results regarding the COx plateau established with ranges based on absolute values (i.e., thresholds of change corresponding to ±5 and ±10 SmO_2_ a.u. for ≥ 30 s) are presented in Table 2.

**TABLE 2:** Critical oxygenation plateau established with ranges based on absolute %SmO_2_ arbitrary units variability (*n* = 120 total, *n* = 30 for each category).

Environment	Muscle	Number of cases with a plateau observed in the last 45 s of the CP test	Percentage of cases with a plateau observed in the last 45 s of the CP test
±5 a.u. range, %SmO_2_			
Normoxia	Vastus lateralis	29	96.66
	Triceps brachii	10	33.33
Hypoxia	Vastus lateralis	28	93.33
	Triceps brachii	20	66.66
±10 a.u. range, %SmO_2_			
Normoxia	Vastus lateralis	29	96.66
	Triceps brachii	22	73.33
Hypoxia	Vastus lateralis	30	100
	Triceps brachii	28	93.33

Abbreviations: CP, critical power; $\%S_{mO_{2}}$, arbitrary unit of muscle oxygen saturation.

Results regarding the SmO_2_ plateau established with ranges based on relative values (i.e., thresholds of change corresponding to ±5 and ±10% of SmO_2_ for ≥ 30 s) are presented in Table 3.

**TABLE 3:** Critical oxygenation plateau by a range based on relative %SmO_2_ variability (*n* = 120 total, *n* = 30 for each category).

Environment	Muscle	Number of cases with a plateau observed in the last 45 s of the CP test	Percentage of cases with a plateau observed in the last 45 s of the CP test
±5% of %SmO_2_ range			
Normoxia	Vastus lateralis	7	23.33
	Triceps brachii	3	10
Hypoxia	Vastus lateralis	7	23.33
	Triceps brachii	4	13.33
±10% of %SmO_2_ range			
Normoxia	Vastus lateralis	15	50
	Triceps brachii	5	16.66
Hypoxia	Vastus lateralis	9	30
	Triceps brachii	12	40

Abbreviations: CP, critical power; %SmO_2_, arbitrary unit of muscle oxygen saturation.

Figure 1 presents averaged SmO_2_ kinetics during the 30 s preceding the CP tests, throughout the tests, and during the 30 s following their completion.

Table 4 presents results for the approach based on independent visual inspection of the individual SmO_2_ curves. The experts reported the frequent occurrence of the plateau in vastus lateralis in both environments (>91% of cases), with moderate to substantial agreement. The non‐experts exhibited poor to substantial agreement in vastus lateralis in both environments (>89% of cases).

**TABLE 4:** Critical oxygenation plateau based on a visual assessment (*n* = 120 total, *n* = 30 for each category).

Conditions	Muscle	By four expert physiologists		By four non‐expert athletes	
		Number of cases with a plateau observed (mean ± SD)	κ value (LoA)	Number of cases with a plateau observed (mean ± SD)	κ value (LoA)
Normoxia	Vastus lateralis	27.5 ± 0.6	0.735 (substantial)	27.0 ± 1.4	0.627 (substantial)
	Triceps brachii	9.8 ± 4.5	0.426 (moderate)	12.8 ± 7.9	0.153 (slight)
Hypoxia	Vastus lateralis	28.0 ± 0.8	0.554 (moderate)	26.8 ± 5.3	−0.068 (poor)
	Triceps brachii	22.8 ± 3.2	0.706 (substantial)	14.3 ± 5.1	0.525 (moderate)

Abbreviation: LoA, level of agreement.

### 3.1 Comparison of the methods

The comparative analysis of the applied methods showed almost perfect agreement between approaches based on a threshold of change of 5 a.u. of %SmO_2_ and expert group visual assessment (93.3%–100% for vastus lateralis and 76.7%–96.7% for triceps brachii). It is noteworthy that non‐expert and expert group visual assessments (the positive agreement of at least three of four raters was included as the confirmation of the plateau) exhibited higher agreement for vastus lateralis (86.7%–90%) than for triceps brachii (50%–70%). Figure 2 includes the case‐by‐case presentation of binary COx plateau determination with different methods.

## 4 DISCUSSION

Based on the analysis of well‐established variables that are associated with some kind of physiological plateau during exercise, different approaches for defining and determining SmO_2_ plateau were proposed. Next, they were applied in a vast dataset presenting NIRS signal curves to verify the existence of COx plateaus during 3 min all‐out cycling trials in different conditions of oxygen availability in vastus lateralis and triceps brachii among highly trained endurance athletes. Determination of the COx plateau depended on the applied methodological approach, with most cases observed in the vastus lateralis in both environments. Methods for determination of the SmO_2_ plateau based on a threshold of change of 5 a.u. of SmO_2_ and expert visual assessment showed almost perfect agreement and are feasible for application in future research and practice.

Given that the quadriceps muscles are the primal movers in cycling, the local strain measured with SmO_2_ remains reliable and exhibits practical value (Feldmann & Erlacher, 2021). Therefore, the frequent occurrence of the COx plateau observed in the vastus lateralis aligns with the mechanistic rationale of the main locomotor muscle as a crucial limiter of peripheral performance (Amann & Dempsey, 2008). The engagement of non‐locomotor muscles, i.e., upper limb and trunk muscles, during cycling is associated with body position (Duc et al., 2008; Turpin et al., 2017). During our study, the athletes could choose their body position freely, meaning that they could shift their mass between various points of support (pedals, seat and handlebars). Such manoeuvres are likely to influence SmO_2_ in the upper limbs and the frequency of occurrence of the COx plateau in triceps brachii. Factors such as standing on the pedals or engaging the upper body to generate higher power were occasionally observed during the last seconds of the effort and could possibly cause a rapid drop in SmO_2_ in muscles not typically associated directly with cycling performance. This could cause high variability in the SmO_2_ curve and reduce the number of detected plateaus.

The approach based on relative percentage changes did not align well with other methods. It is noteworthy that the average level of %SmO_2_ during the plateau was 20.1–22.1 a.u. of %SmO_2_ for females and 10.6%–11.6% a.u. of %SmO_2_ for males. Therefore, the thresholds of change corresponding to 5% or 10% might have been <1 a.u. of %SmO_2_. Such narrow ranges are not feasible during NIRS analysis from wearable devices, owing to natural physiological variability and technical limitations. Given that the steady state in local oxygenation during exercise can occur at different intensities (e.g., at 45 a.u. of %SmO_2_ around the aerobic threshold and at 25 a.u. of %SmO_2_ around the anaerobic threshold), the use of percentage changes exhibits limited universality. Consequently, such an approach should not be applied unless required by a very specific context.

The thresholds of change corresponding to 10 a.u. of %SmO_2_ allowed for to identification of more COx plateaus than the thresholds of change corresponding to 5 a.u. of %SmO_2_. In this regard, it remains unclear whether application of such a wide range leads to false‐positive results (i.e., identifying the plateau when it does not exist). Significant differences between this method and the other methods were observed, with the most noticeable in triceps brachii in normoxia (application of thresholds of change corresponding to 10 a.u. of %SmO_2_ identified 22 plateaus versus 3–10 cases identified with other methods). The observed disparity suggests that the range of ±10 a.u. of %SmO_2_ is too large, especially during high‐intensity exercise, resulting in low %SmO_2_ levels. Consequently, it should not be used for detection of the COx plateau and should be treated with caution when applied in different scenarios.

Visual assessment of the SmO_2_ curve is frequently applied in the literature, and such an approach to determine the SmO_2_ plateau performed by an expert group exhibited almost perfect agreement with the method based on a threshold of change of 5 a.u. of %SmO_2_. The agreement between the individual raters was poor to substantial, exhibiting significantly higher reliability in expert than non‐expert groups. Noteworthy differences between group assessments performed by the experts and non‐experts were observed (the positive agreement of at least three of four raters was included as confirmation of the plateau). These results suggest that high‐level expertise is necessary to identify the plateaus visually. Considering how easy it is to implement, the expert visual method might become prevalent in future research and applied settings. The crucial consideration is the plateau length. We strongly suggest that it should be predefined clearly and presented as a guideline to the raters before their assessment. In addition, it should be documented in the methods section of the publication and, where applicable, incorporated into the study protocol or registered report. Considering the exhibited level of agreement between the expert raters, we recommend employing at least two raters, with a supervisor involved whenever the initial raters do not agree.

### 4.1 Limitations and considerations for praxis

Some limitations of the presented study need to be discussed. First, the Moxy device used in our study, although widely applied in exercise physiology research (Carreño‐Román et al., 2024; Perrey et al., 2024), is not considered a gold‐standard measurement device. However, it was found to produce physiologically credible SmO_2_ values during rest and exercise (McManus et al., 2018). For comparison, another established NIRS monitor called PortaMon (Artinis Medical Systems, The Netherlands) exhibited a smaller dynamic range compared with the Moxy sensor (McManus et al., 2018). This might reduce measured SmO_2_ variability and improve repeatability of the assessment, making identification of the plateau easier and more likely. Therefore, the same approach might produce slightly different results when different NIRS monitors are applied. However, it is not likely to produce significant differences, considering the large dynamic range of the Moxy sensor and repeatability and reproducibility of the results (Feldmann et al., 2019). Moreover, Moxy is a continuous‐wave NIRS monitor and relies on constant differential path length. Therefore, tissue scattering might influence the measurement, and making comparisons between places of the body with different densities or sizes of tissue layers remains challenging. Yet, from a practical standpoint, even wearable and relatively cheap commercial devices (i.e., Moxy) can be used successfully to determine the SmO_2_ plateau during exercise.

The examination of methodological approaches regarding SmO_2_ plateau determination in our study was limited to a 3 min CP cycling test. Given that the test exhibits a power output plateau in the final part, the final segment resembles a quasi‐constant‐load test. Thus, although the visual method of identifying the SmO_2_ plateau was applied successfully in our study, it should not be considered valid within ramp or step tests with short stages without adequate research.

### 4.2 Perspective

The suggested methodology to determine the SmO_2_ plateau should be verified in various efforts (long slow distance and high‐intensity interval training, incremental intensity tests), populations and muscle groups. Additionally, comparison of SmO_2_ responses across different exercise modalities (e.g., cycling vs. running) might clarify the universality of our findings. Moreover, investigation of SmO_2_ kinetics in respiratory muscle remains particularly interesting, because it could shed light on the blood flow interplay between locomotor and non‐locomotor muscles. Further experimental validation should also address synchronized measurements of SmO_2_, blood lactate and electromyography to unravel metabolic, cardiovascular and neural contributions to the plateau phenomenon. Finally, longitudinal studies are also warranted to assess whether SmO_2_ kinetics change with training, providing mechanistic insights into performance gains or lack thereof.

## 5 CONCLUSION

Given that the determination of the SmO_2_ plateau depends on the applied methodological approach, authors should clearly define its criteria in the methods section of their publications. Based on the present work, we suggest application of methods based on a threshold change of 5 a.u. of %SmO_2_ or expert visual assessment, and 30 s segments. A COx plateau was frequently (>90% of cases for the recommended methods) observed in the vastus lateralis in both hypoxic and normoxic conditions during 3 min all‐out cycling trials. Therefore, SmO_2_ kinetics in the main locomotor muscles can be applied to identify COx across different ambient oxygen concentrations.

## AUTHOR CONTRIBUTIONS

Tomasz Kowalski contributed to the conception and design of the work, acquisition, analysis and interpretation of the data, and drafted the manuscript. Kinga Rębiś, Adrian Wilk and Dominika Granda contributed to the data acquisition and revised the manuscript critically for important intellectual content. Andrzej Białecki contributed to the data analysis and revised the manuscript critically for important intellectual content. Tadej Debevec contributed to the conception and design of the work, interpretation of the data, and drafted the manuscript. Raphael Faiss contributed to the interpretation of the data and drafted the manuscript. All authors have read and approved the final version of this manuscript and agree to be accountable for all aspects of the work in ensuring that questions related to the accuracy or integrity of any part of the work are appropriately investigated and resolved. All persons designated as authors qualify for authorship, and all those who qualify for authorship are listed.

## CONFLICT OF INTEREST

None declared.

## Supporting information



Supporting Information

## Data Availability

The anonymized datasets generated and analyzed during the present study are available in the supporting information.
